# Mechanism of Rhinovirus Immunity and Asthma

**DOI:** 10.3389/fimmu.2021.731846

**Published:** 2021-10-06

**Authors:** Zuqin Yang, Hannah Mitländer, Tytti Vuorinen, Susetta Finotto

**Affiliations:** ^1^ Department of Molecular Pneumology, Friedrich-Alexander-Universität (FAU) Erlangen-Nürnberg, Universitätsklinikum Erlangen, Erlangen, Germany; ^2^ Medical Microbiology, Turku University Hospital, Institut of Biomedicine, University of Turku, Turku, Finland

**Keywords:** asthma, rhinovirus, host defense, immune evasion, interferon type I

## Abstract

The majority of asthma exacerbations in children are caused by Rhinovirus (RV), a positive sense single stranded RNA virus of the Picornavirus family. The host has developed virus defense mechanisms that are mediated by the upregulation of interferon-activated signaling. However, the virus evades the immune system by inducing immunosuppressive cytokines and surface molecules like programmed cell death protein 1 (PD-1) and its ligand (PD-L1) on immunocompetent cells. Initially, RV infects epithelial cells, which constitute a physiologic mucosal barrier. Upon virus entrance, the host cell immediately recognizes viral components like dsRNA, ssRNA, viral glycoproteins or CpG-DNA by host pattern recognition receptors (PRRs). Activation of toll like receptors (TLR) 3, 7 and 8 within the endosome and through MDA-5 and RIG-I in the cytosol leads to the production of interferon (IFN) type I and other antiviral agents. Every cell type expresses IFNAR1/IFNAR2 receptors thus allowing a generalized antiviral activity of IFN type I resulting in the inhibition of viral replication in infected cells and preventing viral spread to non-infected cells. Among immune evasion mechanisms of the virus, there is downregulation of IFN type I and its receptor as well as induction of the immunosuppressive cytokine TGF-β. TGF-β promotes viral replication and is associated with induction of the immunosuppression signature markers LAP3, IDO and PD-L1. This article reviews the recent advances on the regulation of interferon type I expression in association with RV infection in asthmatics and the immunosuppression induced by the virus.

## Introduction

Asthma is one of the most common chronic inflammatory diseases, affecting millions of people worldwide. It was reported by World Health Organization (WHO) that asthma affected an estimated 262 million people in 2019 and caused 461,000 deaths (1). The characteristic asthma symptoms are wheeze, cough, and a tight chest caused by lung inflammation and airway narrowing. Many risk factors have been revealed that directly or indirectly affect the development and exacerbation of asthma, including genetic and environmental factors, such as allergens, airway pollution, and viral infection. Human rhinovirus (RV) is a positive-sense single-stranded RNA virus of the picornavirus family. In healthy individuals, it frequently triggers common colds, but has only a slight effect on human health; however, it can contribute to asthma development and exacerbation in children and adults (2, 3). Human rhinoviruses, identified as the common viral type in asthmatic children, have been classified into RV-A, RV-B, and RV-C species, while asthma exacerbations are driven mainly by RV-A and RV-C (4–6).

Rhinovirus infects airway epithelial cells representing the first line of defense, inducing innate and adaptive immune responses. Initially, RV enters into host cell cytosol by receptor-mediated endocytosis, where RV is uncoated and starts replication (7–9). Within the cell RV components, such as dsRNA, ssRNA, viral glycoproteins, or CpG-DNA, are recognized by pattern recognition receptors (PRRs), including toll-like receptors (TLRs), melanoma differentiation-associated protein 5 (MDA-5) and RIG-1-like receptors (RLRs) (10–12), Upon virus entrance, host epithelial cells release various chemokines (e.g., eotaxin, rantes, CXCL10) and cytokines (e.g., IFNs, IL-6, IL-1, GM-CSF) to eliminate virus replication and spread by orchestrating innate and adaptive immune response (13–16). However, chemokine and cytokine releasing patterns vary among people (17).

IFNs have been classified into interferon type I, II, and III based on the structure of binding receptors and established as the most critical antiviral cytokines. In this review, we highlight the role of interferon type I in RV-associated asthma, especially the subtypes IFN-α and IFN-β. Interferon type I are produced by most cell types, while plasmacytoid dendritic cells (pDC) are the main source (18, 19). IFNs’ downstream signaling drives the infected cells into an antiviral status. Moreover, IFNs can also mediate NK cells, dendritic cells, T cells, and B cells to clear viruses.

Although interferon type I works as an effective agent against viruses, the RV also develops its own mechanism to evade the host immune system. Rhinovirus activates TGF-β present in the environment, which in turn promotes viral replication and inhibit effective antiviral immune responses (20, 21).

The mechanism of asthma exacerbation induced by RV and IFNs involved in virus clearance is not entirely understood. In this review, we summarize the recent advances of the literature, expound on some of our views on the regulated expression of interferon type I and their receptor in association with RV infection in asthmatics and the immunosuppression induced by the virus.

## Human Rhinovirus and Its Intracellular Signaling

The Human Rhinovirus (RV) is the most prevalent respiratory virus causing common colds and other upper airway infections in children and adults. Furthermore, it was recently discovered that it can also provoke lower respiratory tract infections, and facilitate the entry of other respiratory pathogens.

RV-infected epithelial cells show a higher susceptibility to respiratory bacteria, such as *Staphylococcus aureus* and *Streptococcus pneumonia*. Epidemiological data show an association between invasive *Streptococcus pneumonia* disease and the seasonality of RV infection (22). *In vitro* studies have shown that disruption of the epithelial barrier facilitates the transmission of bacteria and increases the adhesion and internalization of pathogens (23–25).

Upper airway infections triggered by RV are equally frequent and severe in asthmatics compared to control patients. However, asthmatics are at a greater risk for more severe and long-lasting lower respiratory tract infections (26). The virus can also trigger exacerbations of chronic respiratory disease, such as asthma and COPD. In asthma, around 80% of the exacerbation in children, viruses are detectable and around half of them are associated with the RV (27, 28). Studies show that rhinovirus infections in early childhood can cause wheezing episodes, which are linked to the subsequent development of asthma in the first place. This indicates that rhinovirus infections in the early childhood might be a potential risk factor for the disease development (3, 29–31).

**Figure 1:**
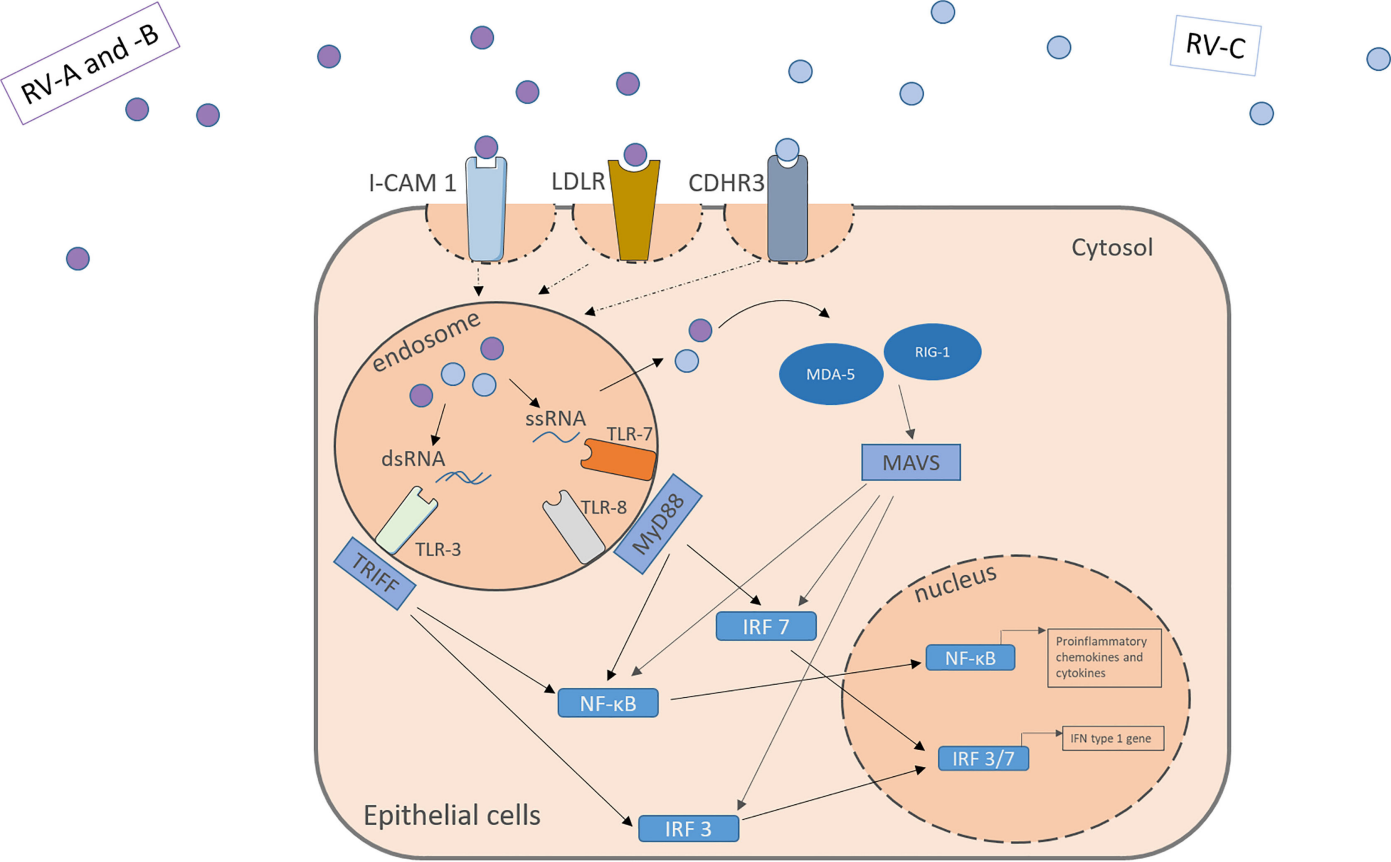
RV activates receptors located apically on the target cell surface. RV-A and –B from the major and minor group activate the ICAM-1 or LDLR, respectively. RV-C enter the cells through CDHR3. Within the cells the virus genome activates pattern recognition receptors in the endosome and the cytosol including TLR3, TLR7, TLR8, MDA-5 and RIG-I leading to the downstream signaling and the translocation of transcription factors into the nucleus. NF-κB and IRF 3/7 bind to specific genes enhances the transcription of proinflammatory cytokines, chemokines and IFN type I genes. IFN, interferon; RV, Rhinovirus; ICAM-1, Intercellular Adhesion Molecule 1; LDLR, Low-Density-Lipoprotein-Receptor; CDHR3, Cadherin Related Family Member 3; dsRNA, double stranded RNA; ssRNA, single stranded RNA; TLR, toll-like-receptor; TRIFF, TIR-domain-containing adapter-inducing interferon-β; MyD88, Myeloid differentiation primary response 88; NF-κB, nuclear factor ‘kappa-light-chain-enhancer’ of activated B-cells; IRF, Interferon regulatory factor; MDA-5, melanoma differentiation-associated protein 5; RIG-1, retinoic acid inducible gene I; MAVS, Mitochondrial antiviral-signaling protein.

The RV is an enterovirus and part of the picornavirus family, and there exist three different species: RV-A, RV-B, and RV-C. Like all non-enveloped viruses, it consists of a capsid with the viral proteins VP1, VP2, VP3 and VP4 and its genome, single-stranded positive-sense RNA, coding for structural and non-structural proteins (32, 33). Depending on the different encoded surface antigens, the RV can be distinguished into 160 serotypes. A possibility to further categorize the different RV-A and RV-B subtypes, is into the minor and the major group, depending on the receptor they use to enter the host cells. While the major group uses the Inter-Cellular Adhesion molecule 1 (ICAM-1, CD54) to enter their target cells, the minor group utilizes the low-density lipoprotein-Receptor (LDLR). The RV-C on the other hand enters the cell through cadherin-related family member 3 (CDHR3) (**Figure 1**) (34, 35).

Those receptors are located apically on the respiratory tract epithelium. Upon activation by the virus, they mediate its uptake through endocytosis. Through structural rearrangement, the uptaken virus can release its genome into the cytosol for replication (7, 9, 35). In the cell, the virus is recognized by pattern recognition receptors (PRR) in the endosome and the cytosol. In the endosome, toll-like-receptor (TLR) 3 recognizes dsRNA and TLR 7 and 8 ssRNA (which is part of the replication cycle of the virus), activating the Myeloid differentiation primary response 88 (MYD88) and TIR-domain-containing -adapter-inducing interferon-β (TRIF) signaling pathways (32, 36, 37). Viruses in the cytosol are recognized by retinoic acid-inducible gene 1 (RIG-1) or melanoma differentiation-associated gene 5 (MDA-5), leading to the activation of the CARDIF signalling pathway (37–39). The joint end of those pathways leads to the translocation of Interferon regulatory factors and nuclear factor ‘kappa-light-chain-enhancer’ of activated B-cells (NF-κB) to the nucleus and eventually to the transcription of antiviral genes (**Figure 1**). Examples for the cytokines released include IL-1, IL-6, IL-8, IL-11, TNF-α, RANTES and granulocyte-macrophage colony-stimulating factor (GM-CSF) and IFN type I and III (13–16). Many of those agents can have pro-inflammatory properties and help the body to clear the virus from the airways, as well as pro viral attributes by helping the virus remain in the tissue.

Besides the released antiviral agents, rhinoviruses also have cytopathic effects on the cells, causing the disturbance of the epithelial integrity and an increase of the epithelial permeability, allowing aeroallergens and other pathogens to migrate into the sub-epithelial tissue facilitating the interaction with the local immune cells (23, 40).

## Interferon Type I and Its Signaling

**Figure 2:**
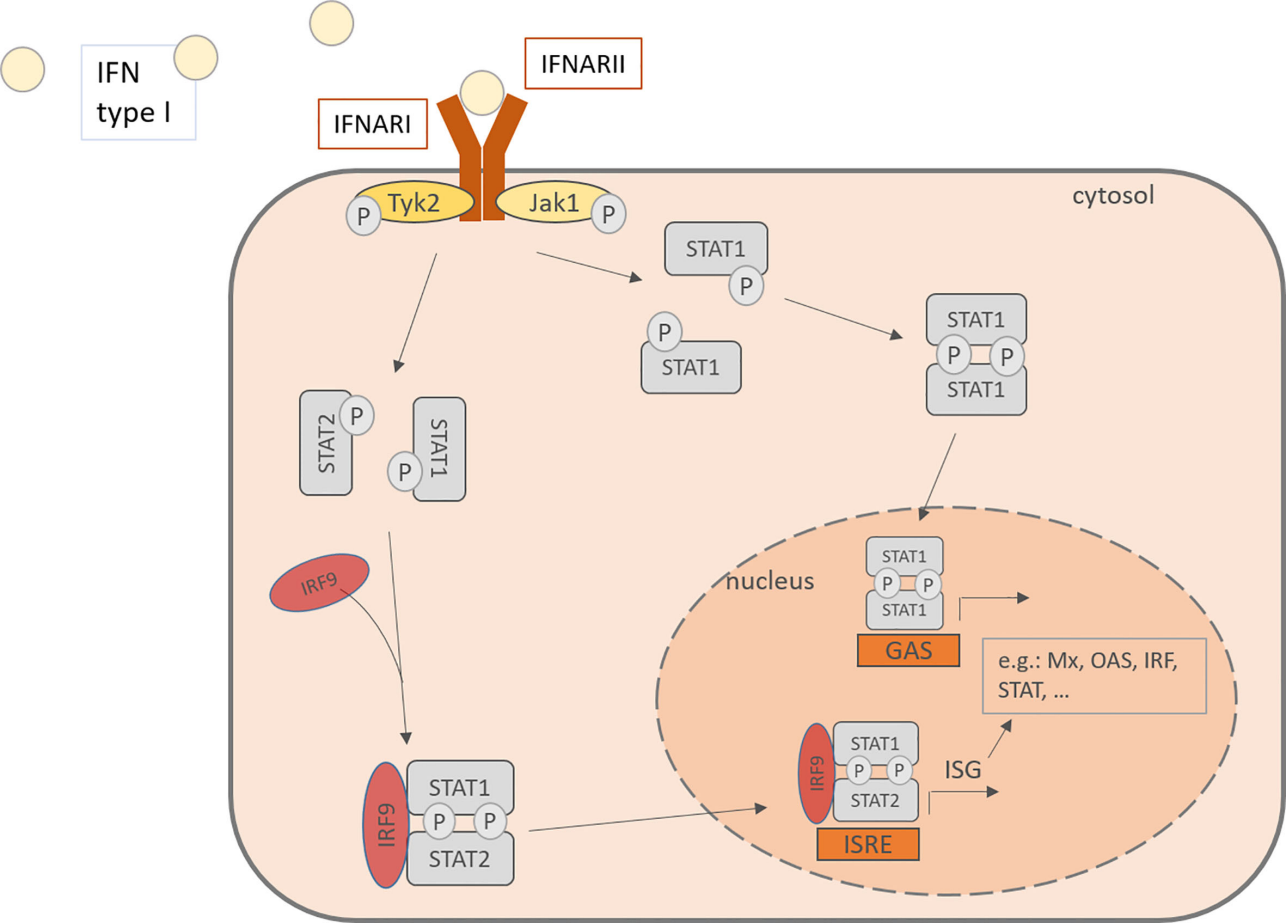
IFN type I activates in an autocrine or paracrine manner the cells through the IFNAR, consisting of IFNARI and IFNARII. This activation of the JAK-STAT pathway leads to the phosphorylation of STAT and its translocation to the nucleus where it binds to promoters and activates the transcription of IFN stimulated genes. IFN, Interferon; IFNARI, Interferon-α/β receptor subunit I; IFNARII, Interferon-α/β receptor subunit II; Tyk2, tyrosine kinase 2; Jak1, Janus kinase 1; Stat, signal transducer and activator of transcription; IRF, Interferon regulatory factor; P, phosphorylated; ISRE, interferon-stimulated response elements; GAS, gamma activating sequences; Mx, Interferon-induced GTP-binding protein Mx1; OAS, 2’-5’-oligoadenylate synthetase.

Interferon type I consists of various subtypes, especially including IFN-α and –β. For IFN-α there exist 13 subtypes and it is expressed mainly by plasmacytoid dendritic cells (pDC), but also by macrophages, monocytes and other virus-infected cells (18, 41). Cellular signaling pathways are activated upon binding of IFN type I to IFN-α/β receptor (IFNAR) which is expressed on most cell types and consists of the IFNAR1 and IFNAR2 subunit. IFNARs are coupled to the Janus kinase 1 (Jak1) and the Tyrosine kinase 2 (Tyk2) and after activation, they phosphorylate signal transducer and activator of transcription (STAT) 1 and 2. The downstream signaling leads to the formation of STAT homodimers and the Interferon-stimulated gene factor (ISGF) 3 complex and their translocation to the nucleus, where they induce the transcription of interferon-stimulated genes (ISG). This induction drives the cells into an antiviral status. ISG include among others OAS1 (2’,5’-oligoadenylate synthetase) and RNase L, both enzymes able to degenerate viral mRNA, PKR, a double-stranded RNA-activates kinase and Mx proteins, a GTPase (**Figure 2**) (42–45). Besides the transcription of antiviral and regulatory genes, the IFN also recruit other cell types that are involved in the defense against viral infections, such as NK cells, dendritic cells, T cells and B cells. Interferon type I monitor the immune system in a real-time and quantitative way. In other words, in host protection the level of IFN type I expression depends on the time after the infection and the amount of RV infecting the host. 24- 48h hours after the infection is the highest peak of viral RNA detectable, while the viral titer was the highest after 24- 72 hours (46).

## Cellular Immunity to RV Infection in Asthma

**Figure 3:**
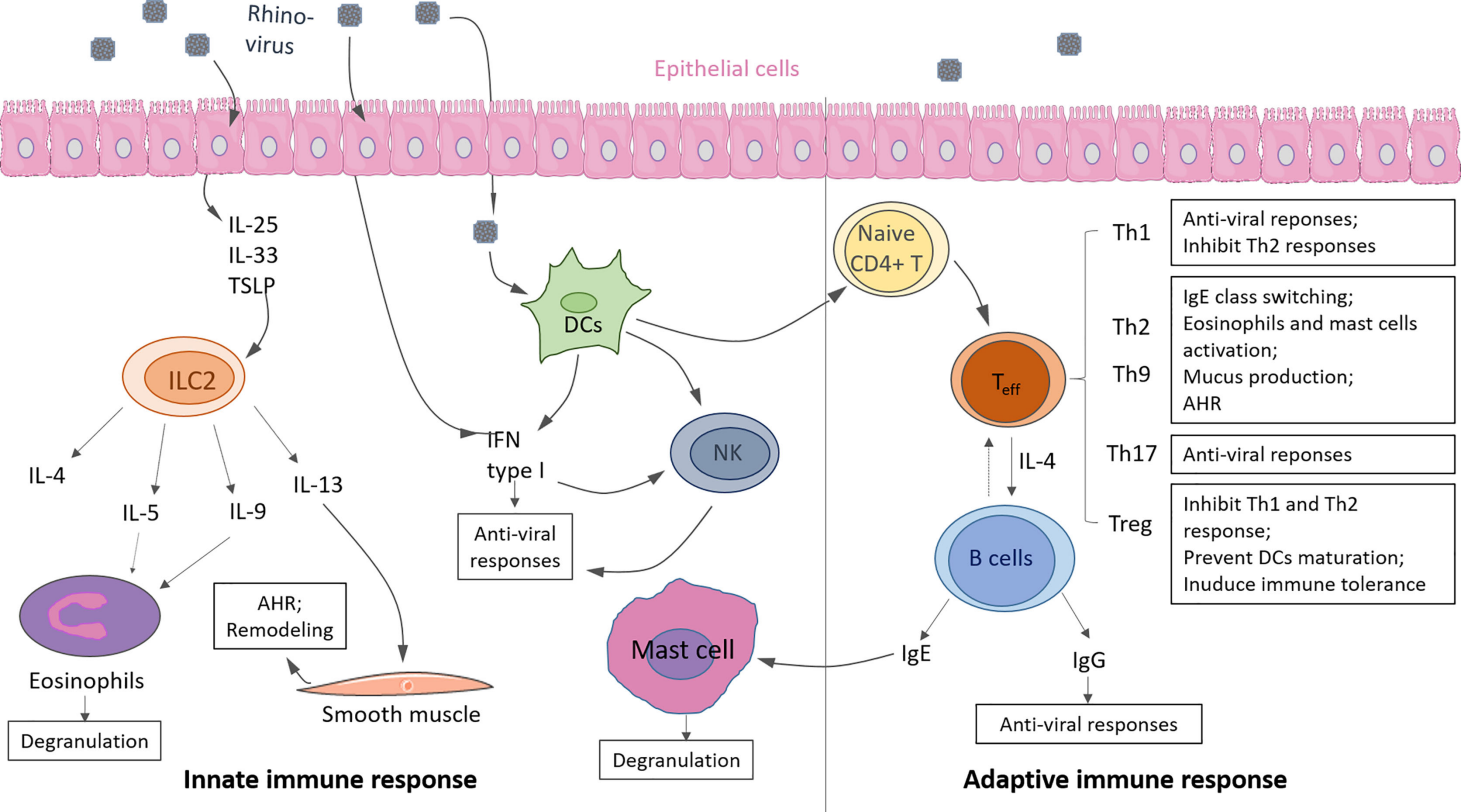
Innate and adaptive immunity in response to rhinovirus (RV) infection in asthma. Innate immunity: Upon RV infection, epithelial cells produce cytokine IL-25, IL-33, TSLP, which promote ILC2 expansion and activation. Type 2 cytokines (i.e., IL-4, IL-5, IL-9, IL-13) are released by activated ILC2, resulting in eosinophils activation and degranulation. Infected epithelium cells, DCs, and DC-activated natural killer cells (NK) produce IFN type I induce anti-viral responses. Adaptive immunity: Activated DCs drive naive CD4+ T cells to differentiate into effector T cells (T_eff_), such as Th1, Th2, Th9, Th17, Treg. T cells can eliminate viruses from the host by releasing anti-viral cytokines or promoting antibody class switching on B cells. IgE causes mast cell activation, and IgG causes viral clearance. Some of the displayed cells were provided by Smart Servier Medical Art (https://smart.servier.com/).

Asthma, a complex disorder, is characterized into Type-2 high and Type 2-low asthma subtypes according to Th2 and ILC2 response levels. Here we update the knowledge about the immunological mechanism of Th2 high asthma and the correlation with RV infection with recent literature. Either hyperactivity of innate or adaptive response can induce Type 2- high asthma. Generally, Type-2 high asthma is accompanied by local accumulation of eosinophils in the airway and hyper-produced IgE, resulting in lung inflammation and airway hyperresponsiveness (AHR) (**Figure 3**) (41).

### Innate Immune Response in Asthma

#### Innate Lymphoid Cells (ILC2)

Type 2 innate lymphoid cells (ILC2) are the main contributors to the innate immunity of Type 2 asthma. When encountering environmental factors, like RV, allergen and pollutants, airway epithelial cells are damaged or dead and as a result they release chemokines (e.g., CCR9, CXCL16) and cytokines (e.g., TSLP, IL33, IL25) also known as alarmins resulting in the recruitment of ILC precursors (ILCP) and drive ILCP differentiation into ILC2 after upregulation of markers like GATA3, retinoic acid receptor–related orphan receptor α (RORα), and T-cell factor 1 (TCF-1) (47). In the murine model of asthma, CXCL16 was demonstrated as the only chemokine triggering ILC2 migration directly, but not the CCL25, a ligand of CCR9 receptor expressed on ILC2 (48). Dependent on IL-33 or IL-25 stimulation, ILCP differentiates into natural ILC2 (nILC2) or inflammatory ILC2 (ILC2), respectively. In asthma, ILC2 producing type 2 cytokine IL-4, IL-5, IL-13, and IL-9 are predominantly induced (49–51). These pro-inflammatory cytokines activate eosinophils and mast cells, and promote IgE class switching on B cells. As a result, ILC2 contributes to asthmatic symptoms in patients with high AHR, damaged lung tissue, and worse lung function. When the alarmin IL-33 is released, it binds ST2 on the ILC2. ST2 is encoded by IL-1RL1 which can undergo alternative splicing generating the soluble decoy receptor (sST2). It has been recently demonstrated that 25(OH)-Vitamin D3 enhances the production of the soluble form sST2. In addition because sST2 neutralizes IL-33 it is considered an anti-inflammatory factor for asthma (52, 53). Moreover, we recently reported that ST2 mRNA was upregulated in the blood cells of asthmatic children with low serum levels of 25(OH)-VitD3. Furthermore, in blood cells from control children and in asthmatic children with RV detected in the airways, the anti-inflammatory isoform sST2 was found to be reduced especially in asthmatic children with low serum levels of 25 (OH)-VitD3. In these control children low levels of 25(OH)-VitD3 correlated with the presence of RV in their airways and with a low IFN-β production in serum (54). Thus these data indicate a counter-regulatory role of 25(OH)-VitD3 on RV induced downregulation of the anti-inflammatory sST2 which can be relevant for future antiviral therapies. A recent study has shown that asthma exacerbations that require corticosteroid treatment can be reduced by vitamin D supplementation in patients with deficient vitamin D levels (55).

Murine studies showed that IFN-γ and IL-1β inhibit ILC2 expansion in RV- induced asthma in baby mice. These data indicate that in RV-induced asthma exacerbation, IL-1β and IFN-γ are two potential therapeutic agents of asthma targeting ILC2 (26, 56). However, further human clinical studies are required to confirm these results in humans.

#### Dendritic Cells

In addition to ILC2, dendritic cells (DC) are another important cell type involved in innate immunity during RV infection. It is well known that DC is a professional antigen presenting cell connecting innate and adaptive immunity. In asthma, DCs drive T cell priming by presenting antigens in the environment (e.g., allergen, virus, air pollution, and so on) to naïve T cells. Based on the function and ontogeny, DCs have been classified into classical dendritic cells (cDC), myeloid dendritic cells (mDCs) and the plasmacytoid dendritic cells (pDCs) subset (57).

Each subtype plays a specific role in innate immunity and affects T cell fate. cDCs derived from a common DC precursor (CDP) are sub-classified into cDC1 (XCR1+CLEC9A+) and cDC2 (CD11b+SIRPa+). Recent studies consider that cDC1 promotes CD8+ T cell priming in mice and Th1 differentiation (57–60). Uniquely producing TLR3, cDC1 might also be involved in antiviral responses (57, 61). cDC2 prime CD4+ T cells and initiate Th2 response. It suggests that cDC2 could be the main dendritic cell subset in asthma (57, 62–64). Similarly, contributing to inflammation, mDCs (CD11b+CCR2+LY6C+CD115+) might be increased in lung in asthma (57, 63). However, more experimental and clinical evidence is required to confirm these findings.

In contrast, pDCs (CD317+SIGLECH+B220+) induce tolerance and maintain lung hemostasis. pDCs produce large amounts of interferon type I against viral infection. pDCs also induce regulatory T cells (Treg) by secreting TGF-β and IL-10, which inhibit Th2 hyperactivity in asthma (65–68). However, in allergic asthma, pDC antiviral responses are reduced by FcϵRI cross-linking with IgE. After exposure to influenza virus, the pDCs from patients with allergic asthma have higher FcϵRIα expression but reduced TLR7 expression and less IFN-α production (69). A subsequent study showed that reduced IFN-α and IFN-λ production correlated with FcϵRI cross-linking (70). Reduced IFN-α and IFN-λ were also found in the allergic asthmatic children exposed to RV compared to control groups (70). Consistently, it was further demonstrated that the interferon-induced antiviral responses *via* pDCs in allergic asthmatic patients could be restored by omalizumab treatment (71). These data suggest that allergen induced FcϵRIα cross-linking impairs interferon responses, promoting RV-induced asthma exacerbation. However, the mechanism of FcϵRI cross-linking induced reduction of interferons on pDC is poorly understood.

#### NK Cells

Also important for the innate immune response and activated by DCs, IL-2, IL-12, IL-15, IL-18, IFN-α and IFN-β are natural killer cells (NK cells). They make up the first line of defense against intracellular pathogens and viruses. They recognize an imbalance of the surface receptors on their target cells, thus detect, and subsequently clear infected cells through their cytotoxic granules containing Granzyme B and through the release of IFN-γ (72). Previous studies showed that the infection with RV16 activates NK cell and modulates their function. This is partly regulated by IFN type I signaling: When IFN type I signaling is blocked, the NK cell degranulation due to RV infection is reduced, as well as the number of activated NK cells (CD107a+) (73).

### Adaptive Immune Response in Asthma

#### T-Cells

CD4+ naïve T cells activated by DCs differentiate into various T cell subsets. These T cells can be grouped into pro-inflammatory and immunosuppressive types. Th2 cells expressing transcription factor GATA3 are thought to be the essential characteristic in asthma. Like ILC2, Th2 cells also produce pro-inflammatory cytokine IL-4, IL-5, IL-9, and IL-13, to promote self-differentiation, activate eosinophils, mast cells, induce airway mucus production and contribute to AHR. Th9 cells are the primary source of IL-9, which maintains mast cell survival and increases mucus production. Th1 cells expressing transcription factor T-box expressed in T-cells (Tbet) are thought of as a friendly cell type in Type 2-high asthma because they inhibit Th2 responses. However, IFN-γ produced by Th1 can induce neutrophils, thus Th1 cells also engage in asthma exacerbation which frequently correlates with Type 2-low phenotype with prominent neutrophils in the lung. Th17 cells producing IL-17A also contribute to lung inflammation in asthma with accumulated neutrophils (74–76). In our study we demonstrated that IL-17A is the target of the RV as the host immune system, during rhinovirus infection, induced IL-17A which inhibits RV infection by downregulating low-density lipoprotein receptor (LDL-R) expression on epithelial cells. Furthermore, in the cohort of preschool children Predicta (Post-infectious immune reprogramming and its association with persistence and chronicity of respiratory allergic diseases) we reported that, in asthmatic children with RV in their upper airways have reduced levels of IL-17A in the supernatants and reduced 2’-5- oligoadenylate synthetase (OAS 1) gene expression in their PBMCs. Of relevance, OAS genes are the first anti-viral defense of the host cells which is activated by interferon type I and it cleave the viral RNA *via* activation of the latent ribonuclease l (RNase L). We also demonstrated that targeted deletion of IL-17A in T cells impaired OAS1 genes downstream of IFN-beta and RV inhibited IL-17A in CD4+ T cells in a setting of asthma. In conclusion our group identified IL-17A as a potent inhibitor of RV1b infection by inducing genes downstream of Interferon type I pathway (77).

#### Treg

Regulatory T cells (Tregs) with transcription factor FOXP3 play an important role in immunotolerance induction in asthma. FOXP3+Treg derive from CD4+ thymocytes (FOXP3+nTreg) or naïve CD4+ cells in the periphery (FOXP3+iTreg) (78). FOXP3+Tregs produce anti-inflammatory cytokines like IL-10 and TGF-β to inhibit hyper Th1 and Th2 responses. Type I regulatory T cells (Tr1) is a subtype of Treg. Although Tr1 does not express FOXP3, it is the main source of IL-10, and can prevent DC maturation *via* CTLA4-CD80/CD86 and LAG3-MHC-II cell contacts, thus results in immunosupressiveness and T cell unresponsiveness (79–82). These data show that FOXP3+Treg cells and Tr1 are crucial inhibitory T cells in maintaining lung homeostasis in asthma.

Clinical studies found that healthy controls have more Treg cells *versus* asthmatic patients. Additionally, FOXP3+Tregs were induced after glucocorticoid (83, 84) treatment in asthmatic patients, suggesting a crucial role of FOXP3+ Treg cells in asthma development and therapy. Moreover, Treg cells are also involved in RV-associated asthma exacerbation. After RV16 infection, Tregs were induced both in healthy control and asthmatic subjects with upregulated anti-viral gene expression, such as *IFI44L*, *MX1*, *ISG15*, *IRF* and *STAT1*. Whereas the Treg cells from asthmatic patients have reduced suppressive capacity against RV-induced lung inflammation and they downregulate gene expression, such as *CTLA4, CD69, NR4A1-3* (85). However, today the studies on the regulatory mechanism of Tregs in response to RV infection are still limited. We don’t know how RV infection impairs the immunosuppressive function of Tregs in asthma, how Tregs mediate the balance between IFNs response and anti-inflammatory responses, and whether there are functionally specific Treg subtypes involved in RV promoting asthma exacerbation.

#### B-Cells

It is well known that the Th2 responses trigger B cell activation and IgE immunoglobulin class switching on B cells in asthma. During the second allergen encounter, IgE binds to its high-affinity receptor FcϵRI on mast cells, basophils resulting in lung inflammation and tissue injury (86).

In addition to T cell-dependent mechanism, B cells act as antigen-presenting cells (APC), and they can be directly activated by taking up allergen. In a house dust mite (HDM) induced murine model, allergen triggered B cell promoted Th2 responses after a second allergen encounter in the lung, but not only in the sensitized of mice (87). However, the underlying mechanism of B cells promoting Th2 expansion is poorly understood at the moment, and studies about RV-infected B cells are largely missing.

B cell also have an effect on innate cells and Th1 cells. As noted above, in addition to mast cells and basophils, FcϵRI is also expressed on DCs and monocytes. It was reported that allergic IgE reduces the antiviral capacity of patients by cross-linking to FcϵRI on pDC, and this reduced antiviral capacity can be restored by omalizumab (69–71, 88). In addition, another study showed that IgE cross-linking also inhibits monocyte maturation and influenza virus-driven Th1 responses, while did not affect Th2 responses (47). These data suggest an effect of B cells on DCs, monocytes and T cell differentiation. However, the underlying mechanisms of IgE cross-linking inhibiting interferon production by pDCs, monocyte maturation and regulating T cell differentiation need further investigations.

## Immune Defense Against RV Infections and Its Immune Evasion in Asthma and Control

Many viruses modulate the immune response in order to survive in their host cells, to be able to replicate further and to infect more cells. To achieve an efficient viral replication the virus must evade the immune system. The different types of viruses have developed various strategies to undergo the body’s defense mechanisms, including variation in their antigen expression as well as a disturbance of the immune response. In the following chapters, we want to describe the advances in the literature regarding the effects and regulation of a number of cytokines and how the RV might interfere or deregulate them. We concentrate especially on the differences known between healthy and asthmatic patients.

### Interferon Type I

A worsening of the respiratory symptoms occurs during asthma exacerbations that can be triggered by respiratory viruses, causing the release of IFN type I. Whether or not the released levels and the course over time differ in asthmatics compared to control patients is currently still a matter of discussion.

Many studies have shown that there are deficiencies in the production of IFN in asthmatic patients, which leaves them with an inadequate immune response to infections. The human bronchial epithelium constitutes the first barrier towards the environment. The two main types of interferons secreted by these cells are IFN-β and IFN-λ. It was found that in asthmatic patients compared to control patients the IFN type I production was delayed (46) or deficient (89). However in those studies the antiviral and proinflammatory immune response remained intact. Sykes et al. (2012) showed that the IFN-α and IFN-β production by cells of the bronchoalveolar lavage (BAL) of asthmatics is decreased compared to control patients. The IFN responses also correlated with the airway hyperresponsiveness and positive skin prick tests (38). An immunohistochemical study on bronchial mucosa biopsies showed similar results (90). The deficient IFN-α and IFN-β production by epithelial cells from asthmatic patients at baseline corresponded to worse symptoms. There was no significant correlation between sub-epithelial cells expressing IFN-α and an improved clinical outcome. However, in acute infection sub-epithelial cells expressing IFN-α correlated significantly with the viral load in the BAL and increased asthma symptoms. Studies analyzing samples from airway epithelial brushings, cultured with RV infection, described consistent results (91). Consistently, a in IFN type I production was observed in patients in which RV was detected *in vivo* compared to patients without RV in their airways. For the latter group the IFN-α detection was significantly lower (19). In preschool asthmatic children, the same deficiency was found during a non-symptomatic phase, however during HRV-induced exacerbations there is a delayed, but sufficient IFN-α production measurable (90, 92). This shown attenuated interferon response was found not only in mild and moderate asthma, but in severe, therapy resistant asthma as well (93). An *in-vivo* examination was provided through experimental intranasal challenge of asthmatic patients with HRV16. Those patients showed a temporal upregulation of IFN-α and IFN-γ levels, which also correlated with the clinical parameters assessed by Cold Symptoms Score (CSS) (94). In pDCs as well as in PBMCs it has been shown that TLR-7/8-agonists, for example Resiquimod, and RV infection lead to an upregulation of IFN type I, however in asthmatic patients, this upregulation was found to be deficient (19, 95). In infants deficiency in IFN type I production in the first place, also caused increased susceptibility to RV infections and thereby to a higher risk of subsequent asthma development (96).

On the other hand, a study comparing rhinovirus-induced type I IFN production in patients with well-controlled asthma did not detect the aforementioned deficiency compared to control patients (97). Also, epithelial cells from asthmatic subjects infected with RSV show a preserved interferon response (98). Studies measuring a range of alternative cytokines including IFN-λ do not show a difference in rhinovirus response between healthy and asthmatic children (99, 100). Finally, patients with non-allergic asthma may show increased type 1 interferon levels (101).

Taken together, the different studies suggest that the results measurable are highly dependent on the subtype of rhinovirus used for the experiment, the asthma disease endotype, the control status of the disease and the time when the samples are taken. This might explain the different results obtained in the recent years (46).

### RV Influences the Release of Various Cytokines

As noted above, in response to rhinovirus infection, other cytokines besides type 1 interferons are released as well, including the pro-inflammatory IL-1b, IL-6, IL-8, IL-11, and IL-12 (99, 100). As a way to evade the immune system, RV inhibits or modulates their release. IL-1b is produced by mononuclear phagocytes as a response to RV and promotes remodeling of the lung and the proinflammatory host response to the virus (42, 102). When it is inhibited by specific antibodies, there is less airway remodeling and smooth muscle proliferation (103, 104). IL-6 was shown to be induced by RV and it works synergistic with TGF-β to induce Th17 differentiation (20, 91). Also inhibited by the RV14 is the release of the Th1-activator IL-12 from mononuclear phagocytes and thereby the usual induction by IFN-γ or LPS is impaired (42, 105).

On the other hand, immunosuppressive cytokines such as IL-10 are released as well. The production and release by mononuclear phagocytes is strongly induced by RV14. This downregulation was also shown in the sputum from virus-induced asthmatics. This leads to an inhibition of the production of cytokines like IL-1, IL-6, IL-12 and TNF-α (42, 105, 106).

#### IL-17A

IL-17A is released by active T-cells and is involved in the induction of neutrophilic inflammation. It was shown that the cytokine modulates the respiratory epithelial response to RV16 infection, synergistically inducing IL-8 and β-defensin production. The effects were not due to alterations of viral uptake and replication. This modulation might support the epithelium in the recruitment of neutrophils, immature dendritic cells and memory T-cells (107).

IFN type I induced JAK-STAT signaling is also modulated by IL-17A. IL-17A amplifies the transduction of the signaling pathway as well as the mucus secretion from goblet cells and thus helps clearing the virus from the airways. It was shown to inhibit RV-A1B replication, while the virus in return also leads to an inhibited transcription of IL-17A in Th17 cells. When less IL-17A is present, the expression of antiviral agents such as OAS1 is reduced leading to better conditions for the survival of the virus (77). In preschool asthmatic children there was shown to be a defect of IL-17A and the downstream signaling of IFN-β. After RV1b infection of PBMC the independency between the IL-17A and IFN-β pathway was shown, since IFN-β was increased, while IL-17A and OAS1 were reduced. Nevertheless, there are hints that IL-17A favors the expression of IFN-β (77).

#### TGF-β

Transforming growth factor beta (TGF-β) is an important cytokine involved in the regulation of airway remodeling and inflammation through its downstream signaling leading to the induction of FOXP3 and RORγ (20). Its functions include the induction of Th17 cells, the recruitment of neutrophils and monocytes and the induction of Tregs (108). Elevated levels of TGF-β2 are associated with severe asthma and during RV infections, it plays an important role for the induction of the viral replication. Blocking of exogenous TGF-β lead to significantly reduced replication of RV-A1B in primary airway epithelial cells (20). At the same time, a decreased expression of Suppressor of Cytokine Signaling (SOCS) 1 and SOCS 3 was found, as well as an increased IFN-β and -λ production. Possibly, because asthmatics have more endogenous TGF-β, the effects measurable were greater (108). Bielor et al. (20) found an upregulation of TGF-β receptor II mRNA levels, as well as the mRNA levels of downstream transcription factors like Foxp3 and RORγ in PBMC challenged with RV. Additionally, the TGF-β in the supernatant was not elevated, while the TGF-β mRNA expression didn’t change, suggesting that RV infection promoted the consume of TGF-β. Furthermore, the expression of the inhibitor latency-associated protein (LAP) and latent TGF-β binding protein (LTBP), two proteins intracellularly interacting with TGF-β, were induced in PBMC from asthmatic children after RV challenge. This suggests that RV in asthmatics leads to the induction of more endogenous TGF-ß and more binding of TGF-β to its receptor on the cell surfaces, thus upregulated TGF-β signaling (20).

After RV infection, TGF-ß promotes viral replication and inhibits IFN-γ-producing Th1 cells (108–110). Therefore, TGF-ß involved in RV-induced asthma exacerbation might enhance viral replication, as well as an inhibition of Th1- mediated antiviral responses. In addition, TGF-β2 not only plays an important role in virus associated asthma exacerbations, but might also be involved in the development of childhood asthma in the first play. TGF-β2 gene polymorphisms are potentially increasing the susceptibility for developing asthma (111).

#### PD-L1

Rhinovirus infection regulates IFN-β/PD-L1 axis in childhood asthma. In a human cohort study, it was recently described that preschoolers suffering from rhinovirus-induces asthma and with reduced forced expiratory volume in 1 second (FEV1) and high serum levels of C-reactive protein (CRP) show increased level of PD-L1 mRNA in total blood cells. In the same cohort was found that the released levels of IFN-β from PBMCs were associated with an induction of PD-L1, however only in the control group (112). In the asthma group, the asthmatic patients with higher IFN-ß level have less PD-L1 expression in total blood cells and better lung function (112). This finding suggests that IFN-ß suppresses PD-L1 expression in asthma, and the improvement of IFN type I expression in PBMCs in pediatric asthma may ameliorate asthma exacerbation. However, the underlying mechanism of the inhibition of PD-L1 by IFN-ß needs to be further investigated in the future.

## Conclusion

In this review, we address the immunity of asthma associated with RV infection and the recent findings from mouse models as well as in clinical data. IFN type I is considered an effective cytokine against viral infection by inducing antiviral gene expression such as ISG and GAS. There are several cells that produce IFN type I and the IFN type I receptor is expressed ubiquitously. Recent studies have already reported the differing patterns of cell activation, function, differentiation, and cytokine secretion between control and asthma patients. However, there is a need for further studies to gain a deeper insight into the interactions between the various immune cell types involved in the pathogenesis of asthma, especially regarding the involvement of the RV. This greater understanding of the underlying mechanisms would open up the avenue to new therapeutic applications in the treatment of asthma and asthma exacerbations.

## Author Contributions

ZY and HM wrote the manuscript. SF and TV revised the final version of the manuscript. All authors contributed to the article and approved the submitted version.

## Funding

This work was supported by a grant, awarded to SF, from the Collaborative Research Centre (CRC) 1181 (Erlangen) (grant number TP-B08 N), German Research Funding (DFG) at the University hospital in Erlangen, Germany. ZY is supported by a CRC1181 grant (grant number TP-B08 N) and HM is supported by the Interdisciplinary Center for Clinical Research (IZKF) at the University Hospital of the University of Erlangen-Nuremberg (MD-Thesis Scholarship Programme "A82"), both awarded to SF in Erlangen.

## Conflict of Interest

The authors declare that the research was conducted in the absence of any commercial or financial relationships that could be construed as a potential conflict of interest.

## Publisher’s Note

All claims expressed in this article are solely those of the authors and do not necessarily represent those of their affiliated organizations, or those of the publisher, the editors and the reviewers. Any product that may be evaluated in this article, or claim that may be made by its manufacturer, is not guaranteed or endorsed by the publisher.
