# Corrigendum: Mechanism of Rhinovirus Immunity and Asthma

**DOI:** 10.3389/fimmu.2021.800020

**Published:** 2021-11-16

**Authors:** Zuqin Yang, Hannah Mitländer, Tytti Vuorinen, Susetta Finotto

**Affiliations:** ^1^ Department of Molecular Pneumology, Friedrich-Alexander-Universität (FAU) Erlangen-Nürnberg, Universitätsklinikum Erlangen, Erlangen, Germany; ^2^ Medical Microbiology, Turku University Hospital, Institut of Biomedicine, University of Turku, Turku, Finland

**Keywords:** asthma, rhinovirus, host defense, immune evasion, interferon type I

In the original article, the authors neglected to include the funder Collaborative Research Centre (CRC) 1181 (Erlangen), grant number TP-B08 N to authors Zuqin Yang and Susetta Finotto.

In the original article, there was an error. The scientific meaning of the following sentence was unclear owing to poor syntax: “In asthma, are predominantly induced ILC2 producing type 2 cytokine IL-4, IL-5, IL-13, and IL-9”.

A correction has been made to **Innate Immune Response in Asthma, Innate Lymphoid Cells (ILC2), Paragraph 1.** The sentence should read: “In asthma, ILC2 producing type 2 cytokine IL-4, IL-5, IL-13, and IL-9 are predominantly induced”.

The same is true of the following sentence: “After RV16 infection, in Tregs were induced both in healthy control and asthmatic subjects with upregulated anti-viral gene expression, such *as IFI44L, MX1, ISG15, IRF* and *STAT1*”.

A correction has been made to **Adaptive Immune Response in Asthma, Treg**, **Paragraph 2**. The sentence should read: “After RV16 infection, Tregs were induced both in healthy control and asthmatic subjects with upregulated anti-viral gene expression, such *as IFI44L, MX1, ISG15, IRF* and *STAT1”*.

The authors apologize for these errors and state that they do not change the scientific conclusions of the article in any way. The original article has been updated.

## Publisher’s Note

All claims expressed in this article are solely those of the authors and do not necessarily represent those of their affiliated organizations, or those of the publisher, the editors and the reviewers. Any product that may be evaluated in this article, or claim that may be made by its manufacturer, is not guaranteed or endorsed by the publisher.

